# A single blastocyst assay optimized for detecting CRISPR/Cas9 system-induced indel mutations in mice

**DOI:** 10.1186/1472-6750-14-69

**Published:** 2014-07-21

**Authors:** Takayuki Sakurai, Satoshi Watanabe, Akiko Kamiyoshi, Masahiro Sato, Takayuki Shindo

**Affiliations:** 1 Department of Cardiovascular Research, Graduate school of Medicine, Shinshu University, 3-1-1 Asahi, Matsumoto, Nagano 390-8621, Japan; 2 Animal Genome Research Unit, Division of Animal Science, National Institute of Agrobiological Sciences, 2-1-2 Kannondai, Tsukuba, Ibaraki 305-8602, Japan; 3 Section of Gene Expression Regulation, Frontier Science Research Center, Kagoshima University, 8-35-1 Sakuragaoka, Kagoshima, Kagoshima 890-8544, Japan

**Keywords:** CRISPR/Cas9, Gene targeting, Microinjection, Blastocyst, T7 endonuclease I, Surveyor assay, Crude DNA solution, Whole genome amplification

## Abstract

**Background:**

Microinjection of clustered regulatory interspaced short palindromic repeat (CRISPR)/CRISPR-associated protein 9 (Cas9)-related RNA and DNA into fertilized eggs is a novel approach for creating gene-modified mice. Blastocysts obtained just before implantation may be appropriate for testing the fidelity of CRIPSR/Cas9-mediated genome editing because they can be individually handled *in vitro* and obtained 3 days after microinjection, thus allowing researchers to check mutations rapidly. However, it is not known whether indel mutations caused by the CRISPR/Cas9 system can be reproducibly detected in embryos. In this study, we assessed the detection of CRISPR/Cas9-induced mutations in embryos.

**Results:**

T7 endonuclease I was more effective than Surveyor nuclease for detecting mutations in annealed fragments derived from 2 plasmids, which contained nearly identical sequences. Mouse fertilized eggs were microinjected with CRISPR/Cas9-related RNA/DNA to examine whether non-homologous end joining-mediated knockout and homologous recombination-mediated knockin occurred in the endogenous receptor (G protein-coupled) activity modifying protein 2 (*Ramp2)* gene. Individual blastocysts were lysed to obtain crude DNA solutions, which were used for polymerase chain reaction (PCR) assays. T7 endonuclease I-based PCR and sequencing analysis demonstrated that 25–100% of the embryos were knockout embryos and 7–57% of the embryos were knockin embryos. Our results also established that crude DNA from a single blastocyst was an appropriate template for Whole genome amplification and subsequent assessment by PCR and the T7 endonuclease I-based assay.

**Conclusions:**

The single blastocyst-based assay was useful for determining whether CRISPR/Cas9-mediated genome editing worked in murine embryos.

## Background

Gene-modified (GM) mice are essential resources for studying gene function, developmental processes, and human diseases [1,2]. The production of GM mice, such as conditional knockout (cKO) mice, requires several time-consuming steps. For example, a cKO vector carrying the gene of interest and a drug-resistant gene must be constructed. The constructed cKO vector is then introduced into the genome of embryonic stem (ES) cells via homologous recombination. The resulting cKO cells are used to produce a chimeric mouse, with which germ-line transmission is achieved via ES-derived germ cells. Finally, offspring with the KO phenotype are obtained after genotyping and breeding.

In early 2013, a novel genome editing method, called the “clustered regulatory interspaced short palindromic repeat (CRISPR)/CRISPR-associated protein 9 (Cas9) system”, was reported. The CRISPR/Cas9 system enables the efficient production of biallelic (homozygous) KO cells/embryos [3]. The system comprises CRISPR-coded RNAs (crRNAs), trans-activating crRNAs (tracrRNAs), and Cas9 endonuclease. It was originally identified as an adaptive immune apparatus in bacteria and archaea for defense against invading foreign plasmids and phages [4-6]. A working unit of the CRISPR/Cas9 complex (comprising crRNA-tracrRNA fusion transcripts as guide RNA (gRNA) and humanized Cas9 protein) was established to enable the system to work effectively in mammalian cells [4-6]. The system efficiently introduces double-strand breaks approximately 3 bp in front of a protospacer adjacent motif (PAM) sequence (NGG), which is downstream of the gRNA target sequence (23 nucleotides; GN(19) + PAM) [6]. When the target sequence is repaired by non-homologous end joining (NHEJ), an insertion and deletion (indel) mutation results. When a construct with homology to the sequences upstream and downstream of the gRNA target sequence, such as a cKO vector, is introduced with the Cas9 protein, homologous recombination (HR) occurs [6,7].

The most attractive aspect of the CRISPR/Cas9 system is the efficient production of biallelic KO offspring through the direct injection of mRNA encoding Cas9 and gRNA(s). Wang et al. [3] demonstrated that 57–100% of injected zygotes were biallelic KO, when fetuses derived from the microinjected zygotes were examined. Several researchers have confirmed these results and shown that a high rate of indel mutations can be achieved using the CRISP/Cas9 system in mice [7-11]. However, a major problem associated with this system is the need to confirm the presence of the indel mutation in the target sequence in order to know whether the samples possess the (c)KO genotype. To this end, DNA from fetuses or newborn mice is frequently assessed by nuclease assays and sequencing analysis [3,7-10,12]. For optimization of the CRSIPR/Cas9-based GM mouse system, examining blastocysts at the pre-implantation stage is desirable because the evaluation can be performed 3 days after microinjection and does not require egg transfer to recipients to produce offspring. Unfortunately, few studies have assessed indel mutations at this stage ([8]; 3–7 blastocysts were used in addition to pup samples), probably because extremely low amounts of DNA are recovered at this stage and the collected DNA is occasionally lost.

In this study, we used a single mouse blastocyst as a genomic DNA source for several mutational assays. To establish a sensitive and reproducible assay for the detection of mutations introduced by CRISPR/Cas9-mediated genome editing, we first checked whether T7 endonuclease I could be used as an alternative to the widely utilized Surveyor nuclease. We next microinjected CRISPR/Cas9-related RNA/DNA into fertilized eggs and prepared an optimized crude DNA solution from developed blastocysts to examine NHEJ-mediated knockout and HR-mediated knockin of the endogenous receptor (G protein-coupled) activity modifying protein 2 (*Ramp2*) gene. In addition, we used whole genome amplification (WGA) technology [13-15], capable of producing microgram quantities of genomic DNA from a very small amount of DNA, to see whether DNA amplified from the crude DNA extract of a single blastocyst provided an appropriate template for polymerase chain reaction (PCR) analysis. Our results will be useful for researchers and technicians who want to analyze indel mutations at the blastocyst stage.

## Results and discussion

### T7 endonuclease I is more effective than surveyor nuclease for detecting indel mutations

**Figure 1:**
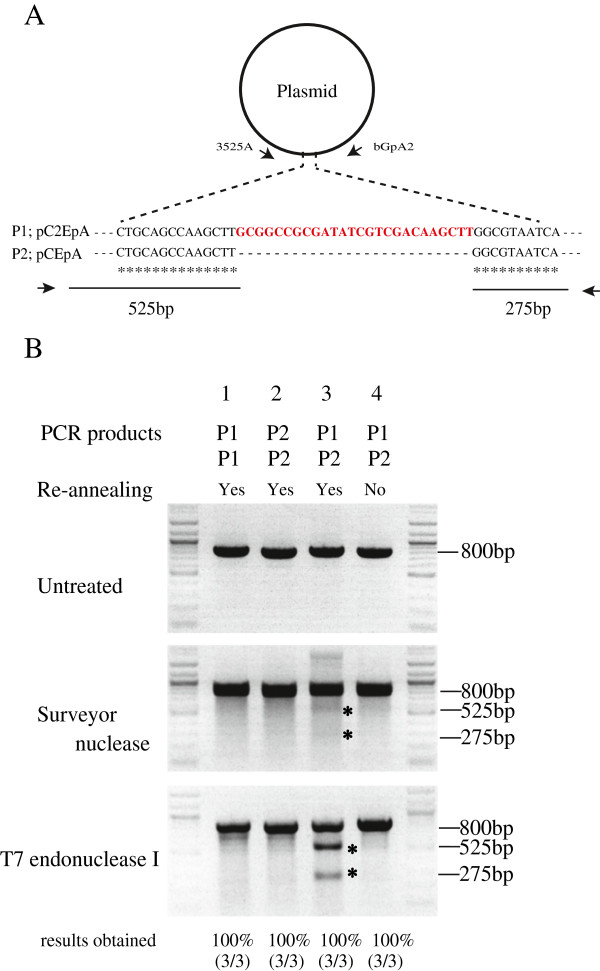
**Comparison of assays using Surveyor nuclease and T7 endonuclease I. A**: P1 and P2 polymerase chain reaction (PCR) products (approximately 800 bp) were used as template to evaluate which nuclease provide clearer and more reproducible results. The P1 and P2 PCR products were generated by PCR of pC2EpA or pCEpA, respectively, using the 3525A and bGpA2 primer set. Both 800-bp products re-annealed were expected to cleavage into two fragments (525 and 275 bp in size) by Surveyor nuclease or T7 endonuclease I. **B**: Agarose gel electrophoresis of nuclease-treated PCR products. A mixture containing P1 (100 ng) and P1 (100 ng) (lane 1), P2 (100 ng) and P2 (100 ng) (lane 2), or P1 (100 ng) and P2 (100 ng) (lane 3) was re-annealed and treated with each nuclease. In addition, a mixture containing P1 (100 ng) and P2 (100 ng) was directly treated with nuclease without re-annealing (lane 4). The 2 asterisks indicate the expected 525- and 275-bp cleavage bands. The experiments were carried out 3 times. The representative results were shown here.

**Figure 2:**
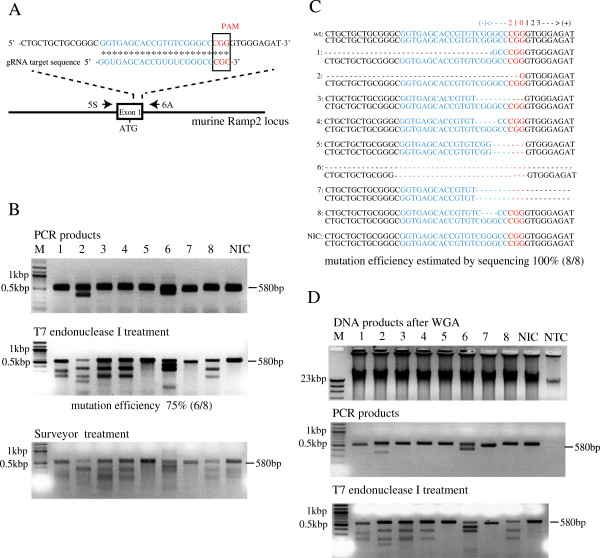
**Detection of CRISPR/Cas9-induced indel mutations in the murine *Ramp2 *gene at the single blastocyst level. A**: Schema of the *Ramp2* guide RNA (R2gRNA) targeting exon 1 in the murine *Ramp2* gene. The R2gRNA-coding sequence is shown in blue. The protospacer-adjacent motif (PAM) sequence is shown in red. The arrows indicate the locations of the PCR primers (see Table 3). **B**: Agarose gel electrophoresis of T7 endonuclease I-treated PCR products and surveyor-treated PCR products derived from 8 individual blastocysts (see Table 1, experiment 3). Lanes 1–8: PCR products amplified from the crude DNA solution of each blastocyst. Lane NIC (no injection control): PCR product amplified from the crude DNA of a single uninjected control blastocyst. M: lambda *Hind*III + 100-bp ladder markers. **C**: Sequencing results for the PCR products shown in lanes 1–8 and NIC of (B). The result of each sample was summarized as the two Ramp2 alleles. “wt” indicates the wild-type sequence. “-” indicates base deletion. **D**: Gel electrophoretic pattern of DNA products after whole genome amplification (WGA) of the crude DNA shown in lanes 1–8 and NIC of (B) (top panel) and after subsequent PCR amplification (middle panel) and T7 endonuclease I digestion (bottom panel). Note that the band pattern is similar to that shown in (B). NTC (no template control) indicates the WGA product obtained using water in place of template as a negative control (top panel); no PCR products were amplified by PCR (middle panel).

To evaluate which nuclease, Surveyor nuclease or T7 endonuclease I, provided clearer and more reproducible results, P1 and P2 PCR products (approximately 800 bp) were used as templates (Figure 1A). The P1 and P2 products were derived from PCR of pC2EpA and pCEpA, respectively, which are nearly identical (Figure 1A). A mixture containing P1 (100 ng) and P1 (100 ng) (lane 1), P2 (100 ng) and P2 (100 ng) (lane 2) or P1 (100 ng) and P2 (100 ng) (lane 3) was re-annealed and treated with each nuclease (Figure 1B). A mixture containing P1 (100 ng) and P2 (100 ng) that had been directly treated with nuclease without re-annealing was used as a control (Figure 1B, lane 4). When the PCR products were subjected to electrophoresis, 2 expected cleavage bands of 525 and 275 bp, generated by nuclease treatment, were observed (asterisks in Figure 1B). Notably, the bands produced by T7 endonuclease I were more distinct than those generated by Surveyor nuclease. The weak cleavage activity of Surveyor nuclease improved slightly when the amount of Surveyor nuclease added to the reaction mixture was increased or the reaction time was extended (data not shown). Furthermore, T7 endonuclease I produced clearer bands than did Surveyor nuclease when the nuclease assay was performed using PCR-amplified products derived from a single blastocyst (Figure 2B, middle and bottom panels). These results suggest that T7 endonuclease I is superior to Surveyor nuclease for detecting indel mutations in target DNA. In the following experiments, we used T7 endonuclease I to detect indel mutations.

### Optimization of crude DNA template preparation from a single blastocyst for highly reproducible direct PCR amplification

To achieve highly reproducible PCR amplification using a single blastocyst as the source of the crude DNA template, we first checked several carriers, including yeast tRNA (Ambion), yeast total RNA (Ambion), herring sperm DNA (Promega), and glycogen (Life Technologies), all of which have been used to precipitate very low amounts of DNA. In preliminary tests, yeast tRNA was better than the other reagents, when evaluated in view of successful PCR amplification (data not shown). Therefore, we used yeast tRNA to prepare a crude DNA solution (10 μL) from a single blastocyst for use in the nuclease assay, as described in the Methods section. We successfully amplified the murine *Ramp2* gene from total 65 individual blastocysts (subtotal 35 in Experiment 1, 2, 3 of Table 1 and subtotal 30 in Experiment 1, 2, 3 of Table 2); see [16] for information on the function of *Ramp2*).

**Table 1:** Detection and efficiency of CRISPR/Cas9-mediated indel mutation at Ramp2 locus at blastocyst stage

**Experiment no.**	**Site injected**^**1**^	**Cas9 mRNA: R2 gRNA (ng/μL)**	**No. zygotes injected**	**No. blastocysts/no. zygotes cultures (%)**	**No. mutant blastocysts/no. blastocysts tested (%)**^**2**^
1	cyto	100:50	25	19/21 (90)	6/21 (32)
2	pn/cyto	100:50	15	8/10 (80)	2/8 (25)
3	pn/cyto	200:50	24	8/17 (47)	8/8 (100)^3^

^1^Injection was performed toward cyto: cytoplasm; pn/cyto pronuclei and cytoplasm.

^2^Mutated blastocysts were identified by both T7EndonucleaseI digestion and sequencing.

^3^Representative results from experiment 3 are shown in Figure 2B and C.

**Table 2:** Dectection and efficiency of CRISPR/Cas9-mediated homologous recombination at Ramp2 locus at blastocyst stage

**Experiment no.**	**Site injected**^**1**^	**Cas9 mRNA: R2 gRNA: R2HR donor DNA (ng/μL)**	**No. zygotes injected**	**No. Blastocysts/no. zygotes cultures (%)**	**No. EGFP-expressing blastocysts/no. blastocysts tested (%)**
1	pn	100:50:10	22	15/22 (68)	1/15 (7)
2	cyto	100:50:10	20	8/16 (50)	2/8 (25)
3	pn/cyto	200:50:10	25	7/20 (35)	4/7 (57)^2^

^1^Injection was performed toward pn: pronuclei; cyto: cytoplasm; pn/cyto: pronuclei and cytopalsm.

^2^Representative results from experiment 3 are shown in Figure 4B and C.

### Use of the single blastocyst-based assay to detect indel mutations introduced by CRISPR/Cas9-mediated genome editing in endogenous murine *Ramp2*

**Figure 3:**
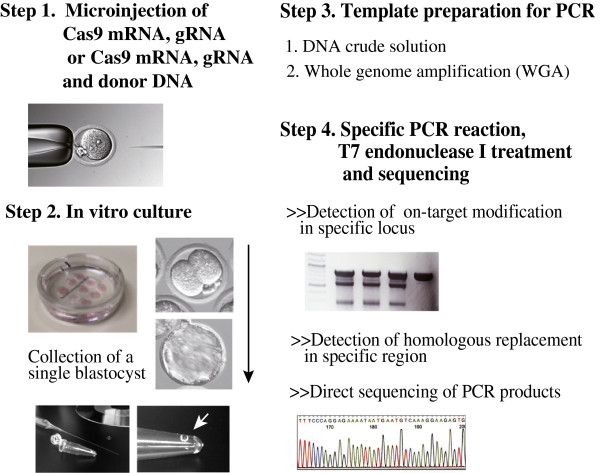
Overview of the nuclease assay for the detection of indel mutations in a single blastocyst.

As shown in Figure 3, we developed a single blastocyst-based mutational assay using T7 endonuclease I and a crude DNA solution. Next, we tested whether the mutational assay could detect mutations in exon 1 of endogenous murine *Ramp2* after microinjection of Cas9 mRNA and *Ramp*2 gRNA (R2gRNA) (Figure 2A) at the zygote stage. Representative results are shown in Figure 2B and C and in Table 1 (experiment 3). First, approximately 2 pL of an RNA mixture (containing 200 ng/μL of Cas9 mRNA and 50 ng/μL of R2gRNA) was injected into the pronuclei and cytoplasm of 24 fertilized mouse eggs (Figure 3, steps 1 and 2). The 17 eggs that survived after injection were cultivated to the blastocyst stage. Crude DNA was then prepared by lysing each of the 8 blastocysts derived from the RNA-injected zygotes (Figure 3, step 3) and 1 blastocyst derived from a non-injected fertilized egg as a negative control (NIC: no injection control). Finally, PCR was performed using each crude DNA sample as template to specifically amplify a 580-bp region corresponding to exon 1 of murine *Ramp2* (Figure 3, step 4). PCR products were successfully amplified from all of the samples tested (Figure 2B, top panel, lanes 1–8 and NIC). Two of the samples produced 2 major bands: unexpected and expected around 580 bp (Figure 2B, top panel, lanes 2 and 6), and 1 of the samples yielded a PCR product slightly shorter than that of the others (Figure 2B, top panel, lane 7). Since Ramp2 has two alleles, these results indicated that the samples in lanes 2 and 6 had relatively long deletions in a heterozygous state or a mosaic state, while the sample in lane 7 had a short deletion in a homozygous state.

To obtain more information on the indel mutations in the 9 samples (Figure 2B, lanes 1–8 and NIC), each PCR product was digested with T7 endonuclease I as described in the Methods section, and the resulting fragments were sequenced (Figure 2C). As expected, the PCR products (580 bp) derived from NIC sample were undigestable. Sequencing of these products revealed no mutations (wild-type (wt) sequence in Figure 2C). PCR products derived from 6 blastocysts (shown in Figure 2B, lanes 1–4, 6, and 8) produced an additional 2 to 4 bands. Sequencing of the PCR product shown in lane 1 revealed a 55-bp heterozygous deletion, located at −58 to −6, where the third nucleotide (G) of the PAM motif (CGG) is considered position 0 (Figure 2C). Similarly, the PCR products in lanes 2, 3, 4, and 8 contained a 206-bp heterozygous deletion (−206 to −1), 10-bp heterozygous deletion (−8 to +1), 4-bp heterozygous deletion (−8 to −5), and 3-bp heterozygous deletion (−7 to −5), respectively. For PCR products in lane 6, both 23-bp deletion (−23 to 0) of two alleles and 372-bp deletion (−244 to +128) of one allele were revealed. The PCR products in lanes 5 and 7 were not digested by T7 endonuclease I; only 1 major band was apparent after nuclease digestion. However, sequencing demonstrated that the samples in lanes 5 and 7 had a 6-bp homozygous deletion (−5 to 0) and 43-bp homozygous deletion (−5 to + 34), respectively. Thus, all of the 8 blastocysts tested had indel mutations in the murine *Ramp2* gene (Table 1, experiment 3). Heterozygous deletions occurred in 6 blastocysts (75%; lanes 1–4, 6, and 8). Homozygous deletions occurred in 3 blastocysts (37.5%; lanes 5–7). These results showed that the T7 endonuclease I-based assay using crude DNA prepared from a single blastocyst is a sensitive and reproducible method for the detection of indel mutations in a target gene.

**Figure 4:**
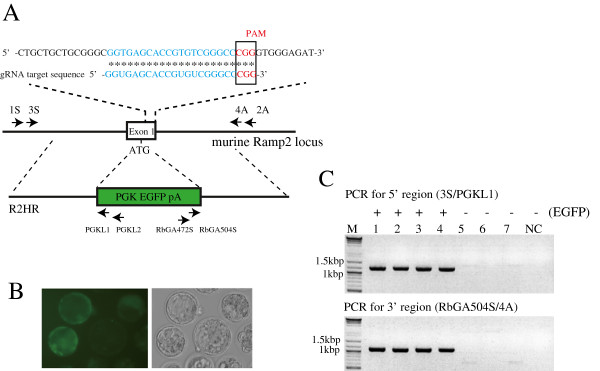
**Detection of CRISPR/Cas9-induced homologous recombination in murine *Ramp2 *at the single blastocyst level. A**: Schema of the R2gRNA and R2HR DNA donor targeting sites in exon 1 of the murine *Ramp2* gene. The R2gRNA-coding sequence is shown in blue. The PAM sequence is shown in red. The R2HR DNA donor comprises 1-kb 5′ and 3′ arms corresponding to exon 1 in the *Ramp2* gene and an EGFP expression cassette. Arrows indicate the locations of the PCR primers (see Table 3). **B**: Fluorescence and bright-field photography of blastocysts developed from fertilized eggs after microinjection (Table 2, experiment 3). **C**: Gel electrophoretic pattern of PCR products corresponding to the junctional region between the 5′ (or 3′) arm and the host genome. Lanes 1–7: PCR products derived from the crude DNA of individual blastocysts. Lane NC: PCR products derived from the crude DNA of a single uninjected control blastocyst. + and –: EGFP-positive and EGFP-negative blastocysts, respectively. Note that PCR products (approximately 1.1 kb) were successfully amplified only in samples from the 4 EGFP-positive blastocysts.

In addition, we tested whether the single blastocyst-based assay could detect an HR event in exon 1 of murine *Ramp2* (Figure 4A) induced by the CRISPR/Cas9 system (Figure 3, step 1). First, approximately 2 pL of an RNA mixture (containing 200 ng/μL of Cas9mRNA, 50 ng/μL of R2gRNA, and 10 ng/μL of R2HR) was injected into the pronuclei and cytoplasm of 25 fertilized mouse eggs. Of these, 20 live eggs were cultivated to the blastocyst stage. Seven embryos developed successfully to blastocysts, of which 4 were EGFP-positive. In Figure 4B, 3 of the 4 fluorescent blastocysts are shown. Next, we prepared crude DNA solutions by lysing each of the 8 blastocysts (4 EFGP-positive and 3 EGFP-negative blastocysts derived from injected fertilized eggs and 1 blastocyst derived from an uninjected fertilized egg). The presence or absence of HR in the target locus was assessed by PCR amplification of the junctional region between the 5′ arm (or 3′ arm) and the host genome (Figure 4A) using crude DNA as template. As shown in Figure 4C, each of the 4 EGFP-positive blastocysts had PCR products with an expected size of approximately 1.1 kb, but the other samples, including the 3 EGFP-negative blastocysts and the 1 uninjected blastocyst, did not. Thus, HR occurred successfully in the 4 EGFP-positive samples, and the HR rate was 57.1% (4/7). These results indicate that single blastocysts are useful for the detection HR events induced by CRISPR/Cas9-mediated genome editing.

### Crude DNA from a single blastocyst is an appropriate template for WGA and subsequent PCR analysis

The main problem when using the T7 endonuclease I-based assay with a single blastocyst is that the DNA content is very small, which often hampers extensive analysis of the target DNA region. For example, we typically prepare a 10-μL crude DNA solution from 1 blastocyst, which is enough for 5 PCR reactions (2 μL/reaction). Although this amount of crude DNA is presently sufficient for analyzing on-target mutations, it is not adequate for examining additional areas to identify possible off-target mutation sites. To resolve this problem, we used WGA, a method for the robust amplification of an entire genome starting with nanogram quantities of DNA [13-15]. When crude DNA solutions prepared from each of the 9 blastocysts (Figure 2B, lanes 1–8 and NIC) were subjected to WGA, 3–4 μg of genomic DNA was successfully amplified (Figure 2D, top panel). When PCR was performed using the DNA generated from WGA as template, a 580-bp product corresponding to exon 1 of murine *Ramp2* was successfully amplified (Figure 2D, middle panel). Notably, the electrophoretic pattern of the PCR products was similar to that of the PCR products obtained from direct amplification of the crude DNA solutions (see Figure 2B, top panel). When the PCR products derived from WGA of the crude DNA were re-annealed and subsequently digested with T7 endonuclease I, the digestion pattern of the PCR products was similar to that of PCR products derived from direct amplification of the crude DNA solution (Figure 2D, bottom panel vs. Figure 2B, middle panel). In the negative control, in which water was subjected to WGA (NTC: no template control), a small amount of DNA was observed, but PCR using the NTC DNA as template did not generate detectable PCR products (Figure 2D, middle panel, lane NTC), as previously suggested by Akasaka et al. [13]. These results indicate that a crude DNA solution prepared from a single blastocyst can serve as template for WGA. Amplification of genomic DNA by WGA may be an effective approach when extensive analyses of on- or off-target mutations are required. However, since WGA has a potential risk of contaminations and the introduction of unwanted mutations [13-15], WGA may require careful evaluation of the results.

Direct microinjection of CRISPR/Cas9-related nucleic acids into zygotes is a powerful tool for the efficient production of GM animals, including mice [3,7-10,12], rats [9,17,18], and monkeys [19]. In most cases, newborn mice or fetuses have been used to confirm the presence of indel mutations in the genome [3,7-10,12]. However, this requires egg transfer to recipients, which is a time-consuming process. The single blastocyst-based assay described here will allow rapid determination of whether the CRISPR/Cas9 system designed by the practitioners is able to generate GM mice, as indicated in Tables 1 and 2. Furthermore, the assay does not require preliminary tests to check the effectiveness of CRISPR/Cas9-related nucleic acids via the transfection of cultured cells. Moreover, if practitioners transfer microinjected zygotes to recipients and do not obtain GM mice, our assay provides a way to determine the reason, for example, fetal lethality caused by a CRISPR/Cas9-mediated mutation or low efficiency of the CRISPR/Cas9-related RNA/DNA.

The present study demonstrated that the crude DNA extracted from a single blastocyst is sufficient for the detection of mutations in nuclease assays that used T7 endonuclease I (see Figure 2) as an alternative to the widely utilized Surveyor nuclease [3,5,7,17]. A few researchers have reported the usefulness of T7 endonuclease I for detecting mutations in a target gene [8-10,18]. Using the single blastocyst assay with T7 endonuclease I and direct sequencing analysis, we demonstrated that microinjection of CRISPR/Cas9-related nucleic acids into zygotes leads to a relatively high degree of mutation (ranging from 25 to 100%; see Table 1), as previously described by Wang et al. [3]. We also demonstrated that the crude DNA sample is sufficient for the detection of HR in the *Ramp2* locus (ranging from 7 to 57%; see Table 2), as previously demonstrated by Yang et al. [7].

Notably, using the present assay and even one experiment with a small number (15–25) of zygotes, researchers and technicians can assess the mutation rate and post-injection embryo survival. This assay also will be helpful to support the animal welfare since the number of mice used in research can be reduced. Furthermore, we demonstrated the usefulness of WGA before PCR and the subsequent nuclease assay (see Figure 2D). WGA amplifies nanogram levels of genomic DNA from a single blastocyst to microgram levels, which enables multiple analyses of genomic DNA, as previously noted by Akasaka et al. [13].

The single blastocyst-based assay described in this study is applicable to the evaluation of mutations generated by the other gnome editing systems, such as zinc-finger nuclease (ZFN) [20,21] and transcription activator-like nuclease (TALEN) [22], because DNA modification through NHEJ and HR with these systems is principally the same as with the CRISPR/Cas9 system.

## Conclusions

We have demonstrated the usefulness of a single blastocyst-based assay for detecting mutations introduced by the CRISPR/Cas9 system. This assay, which is reproducible and convenient, allows researchers and technicians to check the effectiveness of CRSPR/Cas9-based genome editing rapidly.

## Methods

### Vectors and RNA synthesis

The pCEpA and pC2EpA plasmids, which are nearly identical (Figure 1A), were used as PCR templates to evaluate the effectiveness of T7 endonuclease I (New England BioLabs, Japan Inc., Tokyo, Japan) and Surveyor nuclease (Transgenomic Inc., NE, USA) in nuclease assays. To construct the pBS-NFCas9a Cas9 expression vector, cDNA corresponding to the open reading frame (ORF) of hCas9 was first PCR-amplified using phCas9 (#41815; Addgene, Cambridge, MA, USA) as template. Next, the SV40-derived nuclear location signal (NLS) and FLAG sequence were inserted in-frame immediately after the ATG site in the hCas9 cDNA by PCR. The resulting ATG-NLS-FLAG-hCas9 ORF construct was finally subcloned into pBluescript II (Agilent Technologies Japan, Ltd., Tokyo, Japan). For construction of pBS-T7-R2gRNA, the sequence from the gRNA scaffold to the TTTTTT site (83 bp) was PCR-amplified using pgRNA_GFP-T1 (#41819; Addgene) as template and then subcloned into pBluescript II. The resulting plasmid was used as template for inverse PCR. Furthermore, a candidate gRNA target sequence (5′-GGTGAGCACCGTGTCGGGCCCGG-3′) corresponding to exon 1 of the murine *Ramp2* gene [GenBank: NC_000077.6] (Figure 2A) was placed in front of the gRNA scaffold sequence in pBS-T7-R2gRNA. For construction of pR2HR, a genomic DNA sequence of approximately 1 kb (termed “5′ arm”) in front of the ATG site in murine *Ramp2* (Figure 4A) was PCR-amplified using C57BL6/JJcl genomic DNA as template. Similarly, a genomic DNA sequence of approximately 1 kb (termed “3′ arm”) after the ATG site was PCR-amplified. The 5′ and 3′ arms were placed before and after the PGK EGFP pA cassette (containing the mouse PGK promoter, EGFP cDNA, and poly(A) sites) in pBluescript II, respectively. Upon microinjection, the insert containing the R2HR fragment was removed from the vector backbone by digestion with *Not*I and *Sca*I. All of the constructed plasmids (pBS-NFCas9, pBS-T7-R2gRNA, and pR2HR) were sequenced to confirm their fidelity.

Cas9 mRNA was obtained using the mMESSAGE mMACHINE T3 kit (Ambion, Life Technologies Japan, Ltd., Tokyo, Japan) and *Sap* I-linearized pBS-NFCas9 as the RNA synthesis template. R2gRNA (*Ramp2* gRNA) (Figure 2A) was prepared from *Eco*RI-linearized pBS-T7-R2gRNA using the mMESSAGE mMACHINE T3 kit (Ambion).

### Microinjection into fertilized mouse eggs and *in vitro* culture

BDF1 and C57BL6/JJcl (hereafter termed B6) mice were purchased from CLEA Japan Inc. (Tokyo, Japan) and bred with a light period of 7:00 to 19:00. All animal experiments were carried out according to the ethical guidelines of Shinshu University.

Fertilized eggs were collected using a standard *in vitro* fertilization (IVF) method [23]. Briefly, ovulated oocytes were collected from 4- to 10-week-old BDF1 females superovulated by injection with 5 IU of equine chorionic gonadotropin (peamex; Novartis Animal Health Inc., Tokyo, Japan) and 5 IU of human chorionic gonadotropin (Gonatropin; ASKA Pharmaceutical Co., Ltd., Tokyo, Japan) at a 48-h interval and inseminated with B6 spermatozoa obtained from the cauda epididymides of 8- to 10-week-old males. Successfully fertilized eggs were identified by the presence of both pronuclei and the second polar body at 6–7 h after insemination.

Cas9mRNA, R2gRNA, and the R2HR DNA donor fragment were microinjected using a method previously described by Sakurai et al. [23]. Briefly, approximately 2 pL of a 2-template mixture (containing 100 or 200 μg/mL of Cas9mRNA and 50 μg/mL of R2gRNA) or 3-template mixture (containing 10 μg/mL of R2HR, 100 or 200 μg/mL of Cas9mRNA, and 50 μg/mL of R2gRNA) was injected into the pronucleus and/or cytoplasm using a standard microinjection system (Narishige-Olympus MMO-202ND, MM-89, UT-2, IM-9B, and IX-70; Narishige Group, Ltd., Tokyo, Japan). Injected eggs were then cultured in 30 μL of KSOM medium [24] covered with paraffin oil at 37°C in an atmosphere of 5% CO_2_ up to the blastocyst stage. EGFP fluorescence was monitored at the blastocyst stage using an Olympus IX70 inverted fluorescence microscope with the U-MWIBA2 filter set (Olympus, Tokyo, Japan).

### Preparation of crude DNA from a single blastocyst and whole genome amplification (WGA)

Crude DNA derived from a single blastocyst was prepared for use as a PCR template according to the method described by Sakurai et al. [25] with some modifications. Briefly, under a stereomicroscope (SZX12; Olympus), 0.1–0.5 μL of KSOM medium containing 1 blastocyst was transferred to the wall near the bottom of a 0.2-mL PCR tube (thin wall PCR tube with cap; QSP #430-Q; Thermo Scientific, CA, USA) (Figure 3, step 2) using a glass micropipette. Thereafter, 10 μL of blastocyst lysis buffer (125 μg/mL proteinase K, 100 mM Tris–HCl (pH 8.3), 100 mM KCl, 0.02% gelatin, 0.45% Tween 20, and 60 μg/mL yeast tRNA (Ambion)) was gently added to each tube. Each PCR tube was incubated at 56°C for 10 min and then at 95°C for 10 min in a PCR machine (ABI GeneAmp PCR System 9700; Applied Biosystems, Life Technologies Japan, Ltd., Tokyo, Japan). The resulting crude DNA solution was stored at −20°C until use.

For WGA with single blastocyst-derived crude DNA, we used a REPLI-g Mini Kit (Qiagen K.K.,Tokyo, Japan) according to the manufacturer’s protocol. Briefly, 4 μL of crude DNA solution was mixed with 3 μL of Buffer D. The mixture was incubated at 65°C for 10 min, and the reaction was stopped by the addition of 3 μL of stop solution. Finally, 40 μL of REPLI-g solution was added to the mixture, which was incubated at 30°C for 16 h and at 60°C for 3 min.

### PCR

The sequences of the PCR primers are shown in Table 3. A DNA region spanning 800 bp that is different in pCEpA and pC2EpA was PCR-amplified using rTaq DNA polymerase (TaKaRa Bio, Inc., Shiga, Japan) with primers 3525A and bGpA2 (Figure 1A). PCR was performed with 30 cycles of 30 s at 94°C, 30 s at 58°C, and 30 s at 72°C.

**Table 3:** Primer sequences used in this study

**Primers**	**Sequences (5′-3′)**	**Purpose**
3525A	CCGAAGGTAACTGGCTTCAGCAGAGCGCAG	Optimized nuclease treatment (Figure 1)
bGpA2	AACATATGCCATATGCTGGCTGCCATGAAC	Optimized nuclease treatment (Figure 1)
5S	GCCCGAGCTGGAAGCGAGAG	CRISPR/Cas9-mediated mutation (Figure 2A-D)
6A	AAGTAGGAAGCAGCATTAAGT	CRLSPR/Cas9-mediated mutation (Figure 2A-D)
1S	CTGAGTTGTGATAGCTGGCA	CRISPR/Cas9-mediated HR (Figure 4A-C)
3S	AACGTGACCTTAGCCAAGTC	CRISPR/Cas9-niediated HR (Figure 4A-C)
2A	AGTCCAGTTGCACCAGTCCTTG	CRISPR/Cas9-mediated HR (Figure 4A-C)
4A	GTACTCATACCAGCAAGGTAG	CRISPR/Cas9-mediated HR (Figure 4A-C)
PGKL1	GTTGGCGCCTACCGGTGGATGTGGAATGTG	CRISPR/Cas9-mediated HR (Figure 4A-C)
PGKL2	TGTGQGAGGCCAGAGGCCACTIGTGTAG	CRISPR/Cas9-mediated HR (Figure 4A-C)
RbGA472S	TTCCTCCTCTCCTGACTACTCCCAGTCATA	CRISPR/Cas9-mediated FIR (Figure 4A-C)
RbGA504S	CTGTCCCTCTTCTCTTATGAAGATCCCTC	CRISPR/Cas9-mediated HR (Figure 4A-C)

Two rounds of PCR were performed to amplify a 580-bp region of the murine *Ramp2* gene (Figure 2A) using crude DNA as template. For the first round of PCR, a 23-μL reaction mixture containing 1 × Tks Gflex buffer, 0.4 μM each of 2 primers (5S and 6A; Figure 2A), and 1.25 U of a high-fidelity, DNA polymerase derived from *Thermococcus* sp.; Tks Gflex DNA polymerase (TaKaRa Bio, Inc.) was mixed with 2 μL of crude DNA extracted from a single blastocyst. PCR was performed with denaturation at 94°C for 1 min, followed by 30 cycles of 98°C for 5 s, 68°C for 20 s, and 68°C for 5 min. For the second round of PCR, 2 μL of the PCR product from the first round was added to the 23-μL reaction mixture described above. The PCR conditions were the same.

For PCR using DNA derived from WGA, a 25-μL reaction mixture containing 10 mM Tris-Cl, pH 8.3, 1.5 mM MgCl_2_, 50 mM KCl, 0.01% (w/v) gelatin, 0.4 mM of each dNTP, 0.4 μM each of 2 primers (5S and 6A; Figure 2A), 1.25 U of rTaq DNA polymerase, and 50 ng of genomic DNA obtained from WGA was prepared. PCR was performed with denaturation at 94°C for 1 min; 30 cycles of 94°C for 20 s, 58°C for 30 s, and 72°C for 1 min; and a final extension at 72°C for 5 min.

Nested PCR was performed to test whether HR-mediated insertion of the R2HR donor fragment had occurred (Figure 4A). For the first round of PCR, to amplify the 1.1-kb 5′ region of the murine *Ramp2* gene (Figure 4A), a 23-μL reaction mixture containing 1 × Tks Gflex buffer, 0.4 μM each of 2 primers (1S and PGKL2; Figure 4A), and 1.25 U of Tks Gflex DNA polymerase was mixed with 2 μL of crude DNA extracted from a single blastocyst. PCR was performed with denaturation at 94°C for 1 min; 30 cycles of 98°C for 5 s and 68°C for 20 s; and a final extension at 68°C for 5 min. For the second round of PCR, the PCR conditions were the same as described above, except that 3S and PGKL1 were used as primers (Figure 4A), and 2 μL of the PCR solution from the first round was used as template.

The 1.1-kb 3′ region of the murine *Ramp2* gene (Figure 4A) was amplified by 2 rounds of PCR with the same conditions used to amplify the 5′ region of the murine *Ramp2* gene, except that primers RbGA472S and 4A were used for the first round of PCR, and primers RbGA504S and 2A were used for second round (Figure 4A).

### Mutational assays with T7 endonuclease I and surveyor nuclease and direct sequencing

CRISPR/Cas9 -induced indel mutations were detected through mutational assays with T7 endonuclease I and Surveyor nuclease and through direct sequencing of the PCR products. For the Surveyor assay, a crude DNA solution derived from a single blastocyst was purified by ethanol precipitation, dissolved in deionized distilled water, and digested with Surveyor nuclease, according to the manufacture’s user guide (SURVEYOR Mutation Detection Kit for Standard Gel Electrophoresis; Transgenomic). The reaction volume was 11 μL. For the T7 endonuclease I assay, 10 μL of 1× NEB2 reaction buffer (New England BioLabs, Japan Inc.) containing 200–400 ng of PCR product was placed in a 0.2-mL PCR tube (QSP #430-Q; Thermo Scientific). The PCR tube was incubated at 95°C for 5 min for denaturation using an ABI GeneAmp PCR System 9700 (Applied Biosystems, Life Technologies Japan, Ltd.) and then incubated for 0.5–1 h at room temperature (24°C) for re-annealing. Next, 1 μL of T7 endonuclease I (2.5 U/μL) was added to the denatured/re-annealed sample, and the PCR tube was incubated at 37°C for 1–2 h.

The nuclease-treated solutions were electrophoresed using a 2% agarose gel/TAE buffer system, stained with ethidium bromide, and photographed under ultraviolet illumination.

For direct sequencing of the PCR products, 10 ng of PCR product was amplified using BigDye Terminator v.3.1 (Applied Biosystems, Life Technologies Japan, Ltd.) with the 5S or 6A primer and then sequenced with an ABI Genetic Analyzer 3130 (Applied Biosystems, Life Technologies Japan, Ltd.). Sequence analysis was performed using Genetyx-Mac ver.13.0.3 (Software Development Co., Ltd., Tokyo, Japan).

## Abbreviations

CRISPR: Clustered regulatory interspaced short palindromic repeat; Cas9: CRISPR-associated protein 9; PAM: protospacer-adjacent motif; WGA: Whole genome amplification; cKO: Conditional knockout; HR: Homologous recombination; NHEJ: Non-homologous end joining; crRNA: CRISPR-coded RNA; gRNA: guide RNA; tracrRNA: Trans-activating crRNA; ES: Embryonic stem; GM: Gene-modified; *Ramp2*: receptor (G protein-coupled) activity modifying protein 2; NIC: No injection control; NTC: No template control; ORF: Open reading frame; NLS: Nuclear location signal; PCR: Polymerase chain reaction.

## Competing interests

The authors declare they have no competing interests.

## Authors’ contributions

TSa designed the experiments, constructed the plasmids used in the study, performed experiments, drafted the manuscript, and edited the manuscript. AK and TSh assisted in conducting the experiments. SW constructed the plasmids used in the study and edited the manuscript. MS edited the manuscript. All authors read and approved the final manuscript.
